# Expression of hypoxia-inducing factor-1*α* and matrix metalloproteinase-9 in the recipient parasylvian cortical arteries with different hemodynamic sources in adult moyamoya disease

**DOI:** 10.3389/fsurg.2023.1080395

**Published:** 2023-03-14

**Authors:** Mingrui Luo, Jin Yu, Can Xin, Miao Hu, Tianshu Tao, Guiping Wan, Jincao Chen, Jianjian Zhang

**Affiliations:** Department of Neurosurgery, Zhongnan Hospital of Wuhan University, Wuhan, China

**Keywords:** moyamoya disease, intima, cerebral hyperperfusion syndrome, hypoxia-inducing factor-1*α*, matrix metalloproteinase-9

## Abstract

**Objective:**

In our latest research, we have demonstrated that the recipient parasylvian cortical arteries (PSCAs) with hemodynamic sources from the middle cerebral artery (M-PSCAs) has a higher risk of postoperative cerebral hyperperfusion (CHP) syndrome than those from non-M-PSCAs in adult moyamoya disease (MMD) patient. However, whether there are differences between M-PSCAs and non-M-PSCAs in vascular specimens characteristics has not been studied. In this study, we further investigate the vascular specimen of recipient PSCAs by histological and immunohistochemical methods.

**Methods:**

50 vascular specimens of recipient PSCAs were obtained from 50 adult MMD patients during the combined bypass surgeries in our departments of Zhongnan hospital. 4 recipient PSCAs samples were also obtained in the same way from the middle cerebral artery occlusion patients. The samples were received the pathological sectioning, hematoxylin and eosin staining, and immunohistochemistry, then the vascular wall thickness, matrix metalloproteinase-9 (MMP-9) and hypoxia-inducing factor-1*α* (HIF-1*α*) were analyzed.

**Results:**

M-PSCAs adult MMD patients had a thinner intima than non-M-PSCAs in the recipient PSCAs specimens. In recipient non-M-PSCAs vascular specimens, the immunoreactivity indicating HIF-1*α* and matrix metalloproteinase-9 (MMP-9) was significantly higher than M-PSCAs groups. The logistic regression analyses showed that the M-PSCAs was an independent risk factor of postoperative cerebral hyperperfusion (CHP) syndrome (OR 6.235, 95% CI1.018-38.170, *P* = 0.048) in MMD.

**Conclusion:**

Our results indicate that M-PSCAs adult MMD patients had thinner intima than non-MCAs adult MMD patients in the PSCAs. More importantly, HIF-1*α* and MMP-9 were overexpressed in non-M-PSCAs vascular specimens.

## Introduction

Moyamoya disease (MMD) is a chronic cerebrovascular disease that is characterized by bilateral steno-occlusive changes of distal part of internal carotid artery (ICA) and proximal part of middle and anterior cerebral arteries, with abnormal smoke-like vessels at the base of the brain (1). The etiology of the disease is still unknown. MMD occurs around the world, with the most significant incidence in East Asian countries, but rare in Europe and the United States (2).

Cerebral revascularization, superficial temporal artery (STA)–middle cerebral artery (MCA) bypass, is recognized as one of the potent surgical approaches for the treatment of adult patients with MMD (3). STA-MCA anastomosis not only prevents postoperative cerebral ischemic attacks but also reduces the risk of rebleeding in the postoperative period, whereas cerebral hyperperfusion (CHP) syndrome is one of the potential complications of this surgery (4). CHP syndrome is defined as an increase in local cerebral blood flow (CBF) at the anastomotic site of more than 150% over preoperative values, which may lead to intermittent aphasia, epilepsy and even intracranial hemorrhage (5). At the same time, our latest study also found that the recipient PSCAs with hemodynamic source from MCA (M-PSCAs) had a higher risk factor of postoperative CHP syndrome compared to the recipient PSCAs with hemodynamic source from non-MCA (non-M-PSCAs) in MMD (6). Although some studies have proposed differences in the recipient PSCAs (the distal MCA-M4) vascular specimens characteristics of MMD patients, such as, the differences in MMD and cerebrovascular occlusion, adults and children (7, 8), the difference between M-PSCAs and non-M-PSCAs in vascular specimens characteristics in adult MMD has rarely been studied.

In this study, we obtained vessel specimen of the recipient PSCAs (the distal MCA-M4) between M-PSCAs and non-M-PSCAs in adult MMD during surgery. Then, we treated them with histological and immunohistochemical methods. We will study its vascular histopathological and immunohistochemical characteristics to investigate their differences.

## Patiens and methods

### Patients

**Figure 1:**
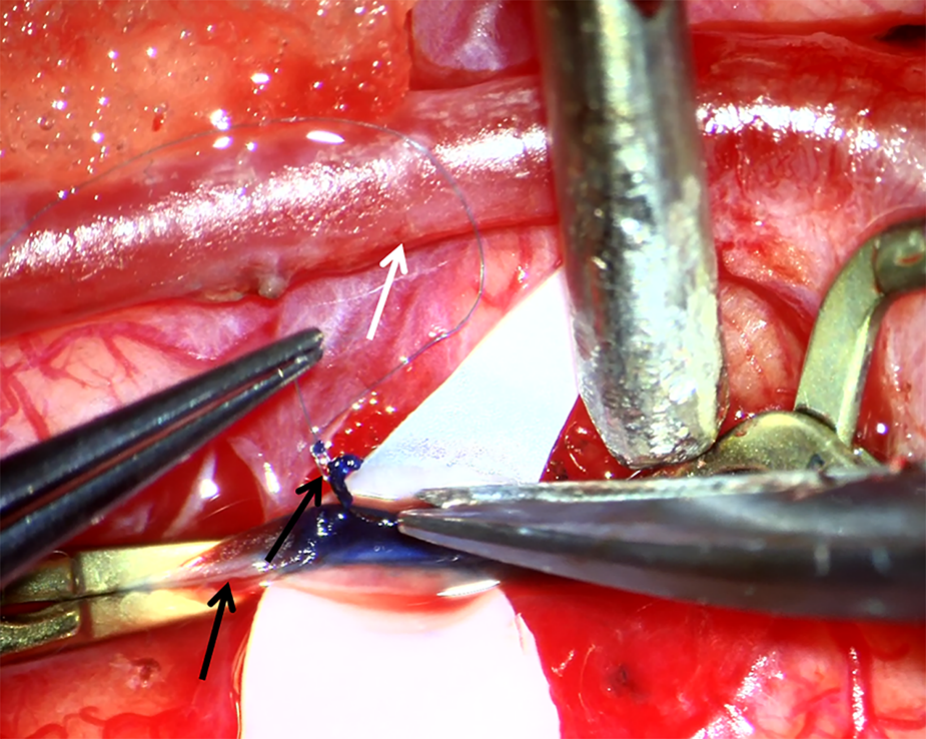
Surgical view of superficial temporal artery (STA)–middle cerebral artery (MCA) bypass. White arrow indicates STA. Lower black arrow indicates MCA (PSCA) and upper black arrow indicates tiny piece of PSCA which is collected as specimen for further histological and immunohistochemical study.

**Figure 2:**
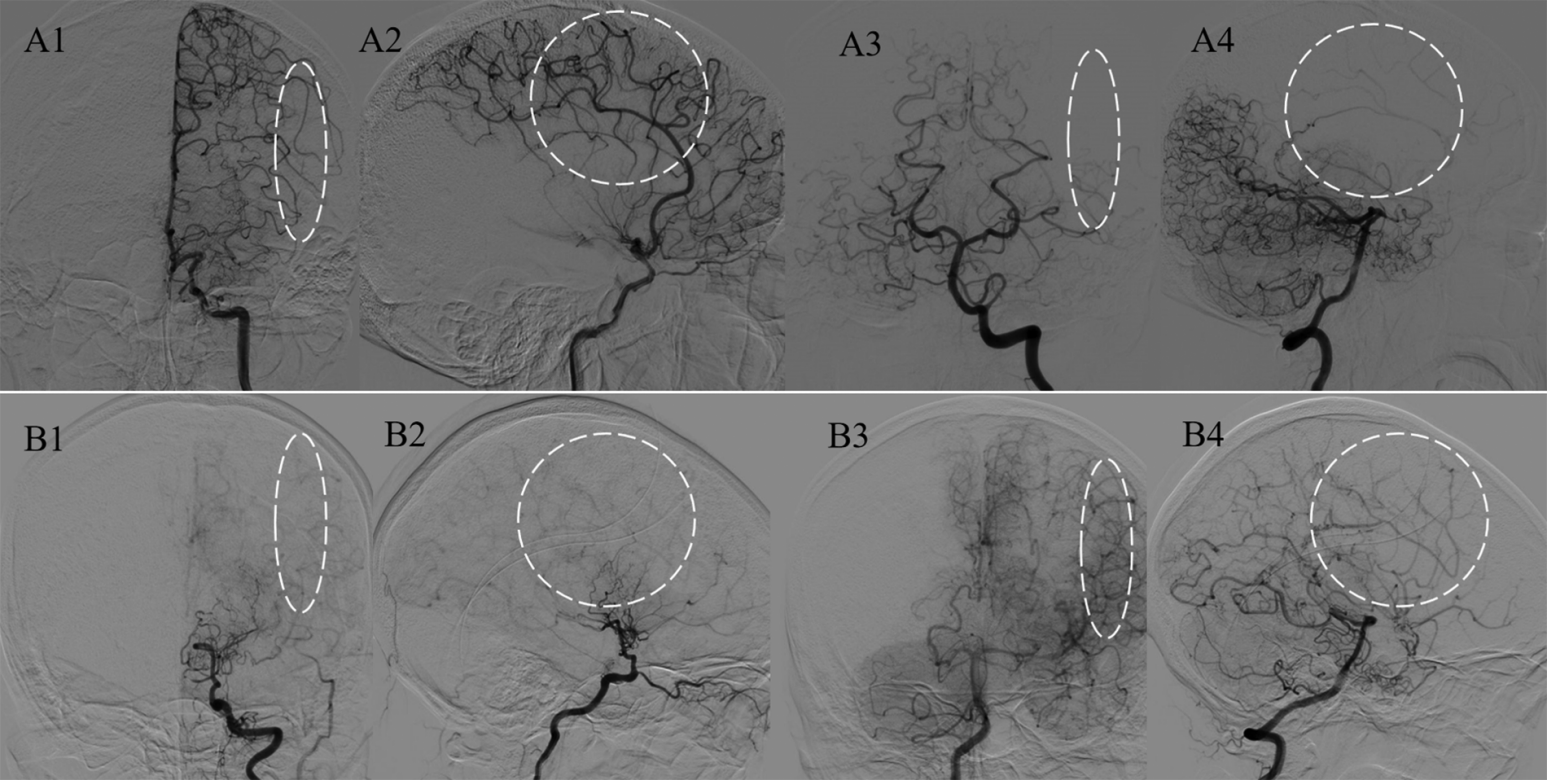
Analysis of the major blood sources of the PSCAs. Representative figures of M-PSCAs and non-M-PSCAs, which are defined as the PSCAs (white circles and ovals) with blood flow from the MCA and non-MCAs. Panels A1–4 indicate the major hemodynamic sources of PSCAs originating from the MCA; B1–4 indicate those from the posterior cerebral artery (PCA).

Fifty patients with MMD were treated surgically at the Department of Neurosurgery, Zhongnan Hospital of Wuhan University, China, from November 2021 to April 2022. All recruited patients were diagnosed as MMD according to the Japanese research committee on MMD (9). We obtained vessel specimens from the MMD patients during the bypass surgery (Figure 1). The grouping of patients with MMD in the preoperative period was blindly investigated by two senior neurosurgeons in preoperative digital subtraction angiography (DSA). According to the hemodynamic source of recipient PSCAs from MCA and non-MCA, the MMD patients were divided into M-PSCAs and non-M-PSCAs (Figure 2). There were 31 people in the M-PSCAs and 19 people in non-M-PSCAs. 4 patients with MCA occlusion were treated with STA-MCA bypass surgery; then four control PSCAs samples were also obtained in the same manner. The baseline data and clinical characteristics of patients were presented in Table 1. This study was conducted under the guidelines provided by Zhongnan Hospital Ethics Committee.

**Table 1:** Characteristics of the patients with moyamoya disease between M-PSCAs and non-M-PSCAs.

	M-PSCAs (*n* = 31)	Non-M-PSCAs (*n* = 19)	*p* value
Age (IQR)	50 ± 15	48 ± 31	0.496
Gender (%)			0.336
Male	19 (61.3)	9 (47.3)	
Female	12 (38.7)	10 (52.6)	
**Tpye of onset (%)**			
Hemorrhage	13 (41.9)	4 (21.1)	0.130
Infarction	11 (35.5)	7 (36.8)	0.923
TIAs	7 (22.6)	8 (42.1)	0.144
**Medical history (%)**			
Hypertension	11 (35.5)	7 (36.8)	0.923
Diabetes mellitus	7 (22.6)	5 (26.3)	0.764
**Life history (%)**			
Smoking	16 (51.6)	8 (42.1)	0.514
Drinking	17 (54.8)	9 (47.4)	0.608
**Suzuki stage (%)**			
3	22 (71.0)	8 (42.1)	0.043
4	7 (22.6)	5 (26.3)	0.764
5	2 (6.5)	6 (31.6)	0.019
Surgical side (%)			0.273
Right	13 (41.9)	11 (57.9)	
Left	18 (58.1)	8 (42.1)	
Postoperative CHP syndrome (%)	12 (38.7)	2 (10.5)	0.031

PSCAs, parasylvian cortical arteries; M-PSCAs, hemodynamic source of PSCAs come form middle cerebral artery; Non-M-PSCAs, hemodynamic source of PSCAs not come form middle cerebral artery. TIAs, transient ischemic attacks; CHP, cerebral hyperperfusion; Data are expressed as mean ± standard deviation. *p* values less than 0.050 were considered significant.

### Sample preparation

In the STA-MCA bypass procedure, a 10-0 nylon monofilament was passed around the wall of the recipient PSCAs (MCA-M4 portion, 0.5–1.0 mm in diameter). By lifting the monofilament with microforces to pull up the PSCAs, the operator carefully dissects the artery using a high-powered microscope. All samples of the PSCAs were obtained postoperatively without disrupting the recipient PSCAs. Then, STA-MCA anastomosis was performed. These samples were first fixed overnight in 10% formalin for several hours and then embedded in paraffin the subsequent day. In each sample, we cut several 4-mm-thick tissue sections from the paraffin block in turn, then removed the paraffin in xylene, rehydrated, and finally received hematoxylin and eosin staining.

### Antibodies

A monoclonal antibody against human hypoxia-inducible factor-1*α* (HIF-1*α*; 1:200; Proteintech, Wuhan, China) and a monoclonal antibody against rabbit matrix metalloproteinase-9 (MMP-9;1:500; Servicebio, Wuhan, China) were performed as the primary antibodies in the present study, respectively. As for secondary antibodies, the horseradish peroxidase (HRP)-conjugated anti-rabbit or anti-mouse immunoglobulin G antibody (1:200; Servicebio, Wuhan, China) were used.

### Immunohistochemical process

In this part, the sections are first washed with phosphate-buffered saline (PBS; PH7.4). After being blocked with 3% H_2_O_2_ and incubating for 25 min at room temperature protected from light, the sections were preincubated with normal goat serum and then incubated overnight at 4 °C with the desired primary antibody. The sections were washed in PBS (pH 7.4). After the sections were slightly dried, the tissue was covered with a secondary antibody (HRP-labeled) of the corresponding species of primary antibody and incubated for several minutes at room temperature. Diaminobenzidine (DAB) chromogenic solution was added to the circles after the sections were slightly dried. The color development time of the sections needs to be controlled under the microscope. The sections were treated with hematoxylin stain solution, hematoxylin differentiation solution and hematoxylin returning blue solution, respectively. The nucleus of hematoxylin stained is blue, and the positive expression of DAB is brownish yellow. Visualize staining of tissue under a microscope (Nikon,Tokyo, Japan) and an image analysis software package (Image-Pro Plus 6.0).

### Immunohistochemical analysis

Under microscope (Nikon,Tokyo, Japan), the histological data were acquired with a computer. Next, the thickness of intima and media and the percentage of immunopositive cell area in the whole specimen were analyzed with Image-Pro Plus. Using Image-Pro Plus, the immunoreactivity was assessed by Luo and Yu blinded to baseline characteristics. They produced their results for the percentage of immunopositive cells in each sample three times and recorded the mean score.

### Statistical analysis

As for the results of the thickness of intima and media and the percentage of the area of immunopositive cells in the whole specimen, statistical analysis was performed using the Mann-Whitney *U* test and Fisher's exact test. The data are were represented as the mean ± standard deviation. The values *p* < 0.05 were considered.

## Results

### Postoperative cerebral hyperperfusion syndrome in moyamoya disease in relation with clinical characteristics

In unitivariate analysis, M-PSCAs, surgical side and hypertension were related to an increased risk of postoperative CHP syndrome, respectively (Table 2). After adjusting for potential covariables (Table 3), the logistic regression analyses showed that M-PSCAs (OR 6.235, 95% CI1.018-38.170, *p* = 0.048) was independent risk factors of postoperative cerebral hyperperfusion syndrome.

**Table 2:** Postoperative cerebral hyperperfusion syndrome in moyamoya disease in relation with clinical characteristics.

Factors	Non - CHP (*n* = 36)	CHP (*n* = 14)	*p* value
Age (IQR)	51 ± 24	48 ± 15	0.888
Gender *n* (%)			0.171
Male	18 (50.0)	10 (71.4)	
Female	18 (50.0)	4 (28.6)	
**Tpye of onset (%)**			
Hemorrhage	11 (30.6)	6 (42.9)	0.410
Infarction	13 (36.1)	5 (35.7)	0.979
TIAs	12 (33.3)	3 (21.4)	0.409
**Medical history (%)**			
Hypertension	9 (25.0)	9 (64.3)	0.009
Diabetes mellitus	11 (30.6)	1 (7.1)	0.082
**Life history (%)**			
Smoking	15 (41.7)	8 (57.1)	0.697
Drinking	15 (41.7)	9 (64.33)	0.151
**Suzuki stage (%)**			
3	24 (66.7)	6 (42.9)	0.123
4	6 (16.7)	6 (42.9)	0.052
5	6 (16.7)	2 (14.3)	0.837
Surgical side (%)			0.019
Right	15 (41.7)	11 (78.6)	
Left	21 (58.3)	3 (21.4)	
Hemodynamic sources (%)			0.031
M-PSCAs	19 (52.8)	12 (85.7)	
Non-M-PSCAs	17 (47.2)	2 (14.3)	

**Table 3:** Multivariate analysis of independent risk factors of postoperative cerebral hyperperfusion syndrome in moyamoya disease.

	OR (95% CI)	*p* value	Adjust OR (95% CI)	*p* value
M-PSCAs	5.368 (1.048–27.502)	0.031	6.235 (1.018–38.170)	0.048
Surgical side	5.133 (1.218–21.630)	0.019	-	0.413
Hypertension	5.400 (1.431–20.382)	0.009	-	0.070

CHP, cerebral hyperperfusion syndrome; PSCAs, parasylvian cortical arteries; M-PSCAs, hemodynamic source of PSCAs come form middle cerebral artery; Data are expressed as mean ± standard deviation; *p* values less than 0.050 were considered significant.

### Thickness of intima and media

**Figure 3:**
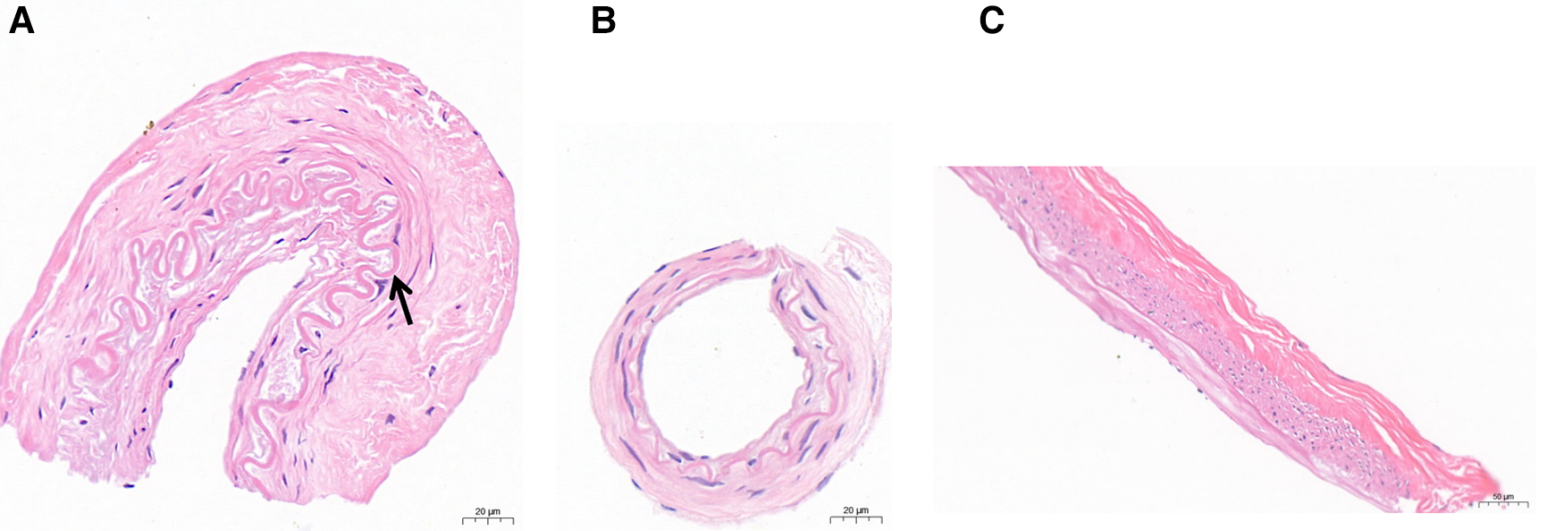
(**A,B**): Photomicrographs of specimens of the PSCAs (MCA) taken from patients with moyamoya disease between non-M-PSCAs (**A**) and M-PSCAs (**B**) disease showing intimal hyperplasia and curved internal elastic lamina (**A**, arrow), and specimens of the PSCA (**C**) taken from control subjects. Scale bar, 20 *µ*m (**A,B**) and 50 *µ*m (**C**); Hematoxylin and eosin stain, original magnification, ×40 (**A,B**) and ×20 (**C**).

**Figure 4:**
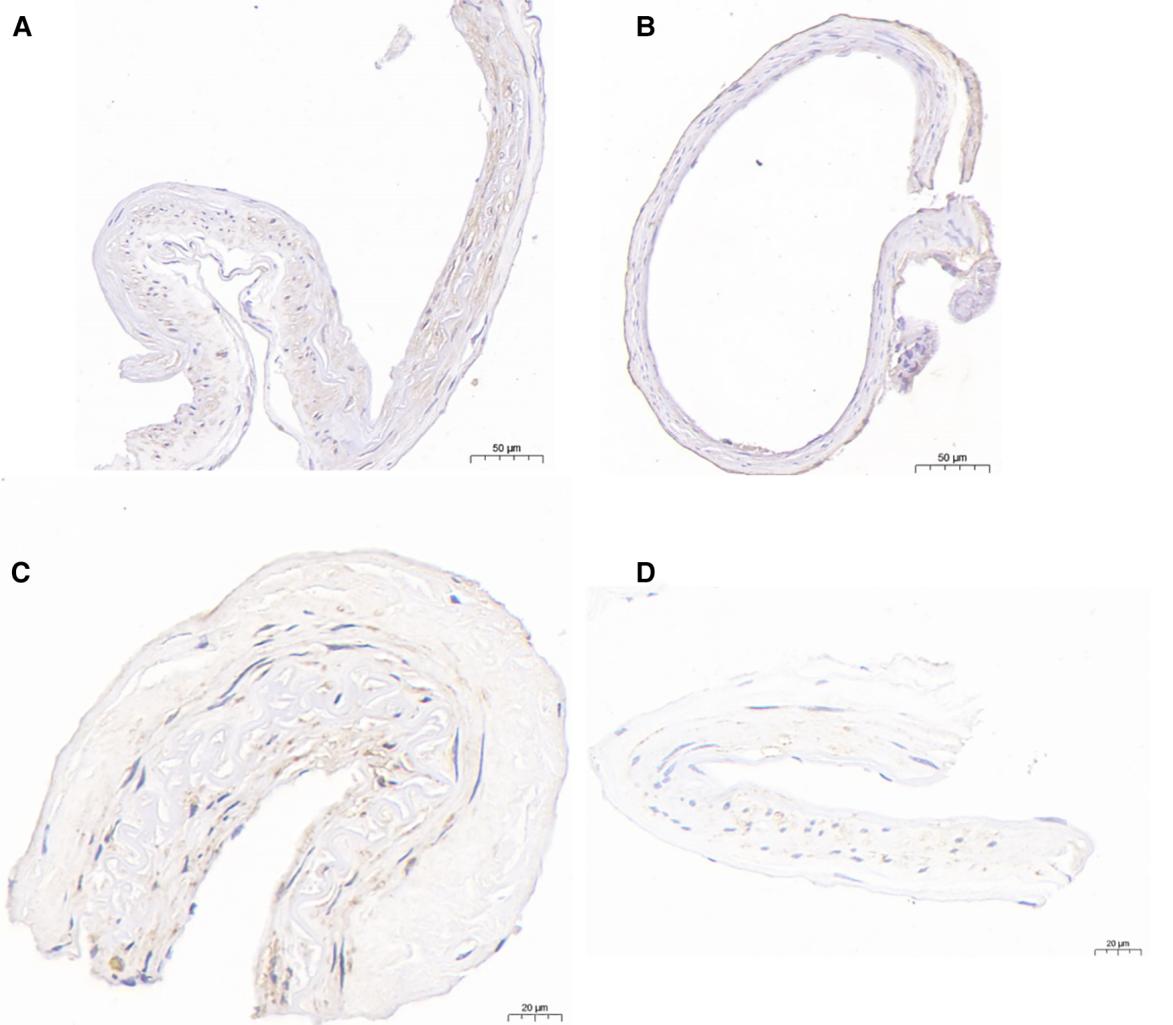
Immunohistochemical analysis of HIF-1*α* and MMP-9.HIF-1*α*-immunopositive cells were detected in the PSCAs specimens from patients with non-M-PSCAs MMD (**A**). (**B**), in M-PSCAs MMD, a smaller number of such cells are detected. MMP-9-immunopositive cells were detected in the PSCAs specimens from patients with non-M-PSCAs MMD (**C**). (**D**), in M-PSCAs MMD, a smaller number of such cells are detected. Scale bar, 50 *µ*m (**A,B**) and 20 *µ*m (**C,D**); original magnification, ×20 (**A,B**) and ×40 (**C,D**).

With the above sections, the thickness of the intima and media in hematoxylin and eosin-stained were measured (Figure 3). The vascular walls of PSCAs from MMD had thicker intima compared to the control recipient PSCAs (MCA occlusion). In contrast, media became thinner in MMD patients. The mean thickness of intima in MMD patients was significantly higher than that in MCA occlusion (Figure 4A–C, Table 4: MMD, 14.2 ± 4.2 *μ*m; and MCA occlusion, 10.0 ± 1.4 *μ*m, *p* = 0.024), but the mean thickness of the MMD media was obviously thinner (Figure 3A–C, Table 4: MMD, 27.6 ± 7.3 *μ*m; and control, 40.5 ± 8.3 *μ*m; *p* = 0.006). Subsequently, we performed an analysis of the thickness of intima and media between recipient M-PSCAs and non-M-PSCAs in MMD patients. In the present study, there was a significantly higher intimal thickening in non-M-PSCAs than M-PSCAs in MMD patients (Table 5: M-PSCAs, 12.8 ± 3.8 *μ*m; non-M-PSCAs, 16.5 ± 4.0 *μ*m, *p* = 0.004). However, there was no difference between M-PSCAs and non-M-PSCA in MMD in the media thickness. (Table 5: M-PSCAs, 26.4 ± 7.2 *μ*m; non-M-PSCAs, 29.5 ± 7.3 *μ*m, *p* = 0.087).

**Table 4:** Intimal and media thickness of middle cerebral arteries of patients with moyamoya disease.

	MMD (*n* = 50)	Control (*n* = 4)	*p* value
Intimal thickness (*μ*m)	14.2 ± 4.2	10.0 ± 1.4	0.024
Media thickness (μm)	27.6 ± 7.3	40.5 ± 8.3	0.006

MMD, moyamoya disease; Data are expressed as mean ± standard deviation. *p* values less than 0.050 were considered significant.

**Table 5:** Intimal and media thickness of middle cerebral arteries between M-PSCAs and non-M-PSCAs in moyamoya diseasea.

	M-PSCAs (*n* = 50)	Non-M-PSCAs (*n* = 12)	*p* value
Intimal thickness (μm)	12.8 ± 3.8	16.5 ± 4.0	0.004
Media thickness (μm)	26.4 ± 7.2	29.5 ± 7.3	0.087

Data are expressed as mean ± standard deviation; *p* values less than 0.050 were considered significant.

### Expression of HIF-1*α* immunoreactivity

Subsequently, an immunohistochemical studies on the recipient PSCAs in MMD patients with HIF-1*α* were performed. The expression of HIF-1*α* in non-M-PSCAs is higher than M-PSCAs. For the M-PSCAs specimens, 37.1 ± 13.8% of the area of immunopositive cells demonstrated anti-HIF-1*α* immunoreactivity in the whole specimen (Figure 4, Table 6). On the contrary, for non-M-PSCAs specimens, the values were 49.2 ± 16.4% (Figure 4, Table 6). (*p* = 0.041).

**Table 6:** Expression of hypoxia-inducing factor-1*α* and matrix metalloproteinase-9 in middle cerebral arteries between M-PSCAs and non-M-PSCAs in moyamoya diseasea.

	M-PSCAs (*n* = 50)	Non-M-PSCAs (*n* = 12)	*p* value
HIF-1*α* (%)	37.1 ± 13.8	49.2 ± 16.4	0.041
MMP-9 (%)	18.2 ± 7.0	25.6 ± 7.7	0.020

HIF-1α, hypoxia-inducing factor-1α; MMP-9, matrix metalloproteinase-9.

Data are expressed as mean ± standard deviation; *p* values less than 0.050 were considered significant.

### Expression of MMP-9 immunoreactivity

Finally, we also did an immunohistochemical studies on the recipient PSCAs in MMD patients with MMP-9. As regards the M-PSCAs MMD specimens, 18.2 ± 7.0% of the area of immunopositive cells demonstrated anti-MMP-9 immunoreactivity in the whole specimens (Figure 4, Table 6). On the contrary, for non-M-PSCAs MMD specimens, the values were 25.6 ± 7.7% (Figure 4, Table 6). (*p* = 0.020.).

## Discussion

In this study, we have access to data on the recipient PSCAs specimens from the patients during surgery. With histopathological findings, MMD patients had thicker intima and thinner media than MCA occlusion, but M-PSCAs MMD patients had a thinner intima than non-M-PSCAs. In addition, the immunoreactivity of HIF-1*α* and MMP-9 in whole specimens was higher in non-M-PSCAs MMD than that in M-PSCAs MMD patients.

In the histological investigations of autopsy specimens, we have known that the MCA from MMD patients had fewer than normal smooth muscle cells both in the media and thick intima (10). This study also found such results in the recipient PSCAs (M4 portion) when MMD was compared to MCA occlusion. It may indicate that the lesions of the whole cerebrovascular was similar in MMD. Meanwhile, the M-PSCAs had a thinner intima than non-M-PSCAs in adult MMD patients. During bypass surgery, our senior neurosurgeons found that the recipient M-PSCAs vascular walls in MMD are transparent and thinner, which meant that it was prone to tearing when anastomosing it, leading to longer operation times and narrowing of the anastomosed vessels. So, it's a challenge to some neurosurgeons. Combined with the study, M-PSCAs in MMD patients was independent risk factors of postoperative cerebral hyperperfusion syndrome (CHP), because it may lead to that intracranial hemodynamic distribution after surgery will be limited to an extremely fixed zones (11). Furthermore, our recent work also found that M-PSCAs in MMD patients were significantly correlated with the onset of focal CHP because the blood supply of M-PSCAs usually came from a morbid MCA, which are severely stenotic or smoke-like vessels, but majority of the another group had normal hemodynamic source, leading to lower blood flow velocity, pressure and postoperative hemodynamic changes in M-PSCAs (6). In addition, the formation of smoke-like vessels in M-PSCAs may be unstable and the cerebral cortex perfused by each artery may be more restricted in a certain area in M-PSCAs compared to that in non–M-PSCAs MMD patients (6). Postoperative CHP syndrome is one of the serious complications after direct bypass that result in intermittent aphasia, epilepsy, and even intracerebral hemorrhage (12, 13). Therefore, whether the recipient M-PSCAs in MMD patients are suitable for direct bypass should be further discussed. In future work, we may do a certain number of indirect bypasses and combined bypasses in M-PSCAs MMD patients, followed by follow-up to compare the efficacy of both procedures. Finally, we will identify the role of direct bypass in M-PSCAs MMD and determine whether it needs direct bypass.

Why there are significant differences in intimal thickness between M-PSCAs and non-M-PSCAs in MMD has confused us. According to that the blood supply of M-PSCAs usually came from non-normal MCA compared to non-M-PSCAs, we have some speculations. On the one hand, owing to the blood supply of it from stenotic MCA and the self-protective mechanism in the brain, PSCAs will increase cerebral blood flow by dilating the diameter of blood vessels. On the other hand, that the blood supply to M-PSCAs comes from severely stenosed or smoke-like MCAs leads to a change in blood flow volume through the stenosed vessels, which may increase the pressure on the vessels. Within the study, we also performed an immunohistochemical analysis of HIF-1*α* and MMP-9 expression in the recipient PSCAs vascular specimens. HIF-1*α* is a major factor participating in tissue-oxygen homeostasis and vascular endothelial growth factor (VEGF); MMP-9 also involves VEGF. Therefore, we chose HIF-1*α* and MMP-9 because they are genes related to VEGF. We all know that MMD is a chronic as well as ischemic cerebrovascular occlusive disease. HIF-1*α*, a transcriptional activator participating in tissue-oxygen homeostasis, is a heterodimeric protein formed by inducible HIF-1*α* and constitutively expressed HIF-1*α* proteins (14, 15). In periods of cerebral ischemia, HIF-1*α* is activated due to insufficient oxygen supply and a decrease in the partial pressure of oxygen in the tissues (16). In the assistance of co-activators, such as cyclic adenosine monophosphate response element-binding protein and acetyl-transferase, both HIF-1*α* and HIF-1β form a heterodimer (17) and HIF-1*α* is moved to the nucleus and binds to the target gene hypoxia response element (HRE), followed by induction of downstream gene expression. Its target genes encode molecules participating in many important physiological activities, such as vasomotor control, angiogenesis, erythropoiesis, cell proliferation, energy metabolism, and so on (18–20). VEGF is a renowned downstream target gene of HIF-1*α* and widely involved in the pathological process of cerebral ischemia, and the VEGF family is distinguished by its powerful angiogenic properties (21). In our present study, we found that the expression of HIF-1*α* in non-M-PSCAs is higher than M-PSCAs in vascular specimens. That would explain why M-PSCAs MMD had a thinner intima compared to non-M-PSCAs. At the same time, the study also found that HIF-1*α* is expressed in the chronic hypoxic zone surrounding the infarcted area during cerebral ischemia (22). Therefore, HIF-1*α* may be a valuable new therapeutic target, especially for ischemic MMD.

In present study, we also found that Suzuki stage 3 and 5 were an independent risk for M-PACSAs and non-M-PSCAs, respectively (Table 1). It indicates that there may be a temporal correlation between M-PACSAs and non-PSCAs in disease progression. In other words, M-PSCAs may transform into non-M-PSCAs with the time going by. In our study, we also analyzed the expression of MMP-9 in vascular specimens of non-M-PSCAs and M-PSCAs by immunohistochemistry. MMP-9 is named gelatinase B and its substrates include gelatins (denatured collagens), native type IV, V and XI collagens, laminin and so on (23). MMP-9 has a dual function when involved in angiogenesis (24). In the early phase of cerebral angiogenesis, the increased expression of MMP-9 under the regulation of vascular endothelial growth factor leads to the disruption of endothelial junctions and the release of hidden VEGF binding sites (25–27). In our study, the expression of MMP-9 in non-M-PSCAs is higher than M-PSCAs in vascular specimens, which further demonstrates why non-M-PSCA has a thicker intima compared to M-PSCAs and that M-PSCAs may transform into non-M-PSCAs in the future. In the later phase of cerebral angiogenesis, excessive activation of MMP-9 exhibits anti-angiogenic effects by degrading endothelial basal lamina and tight junction proteins, leading to endothelial instability and eventually causing blood-brain barrier (BBB) breakdown (23). A study also revealed that melatonin inhibits MMP-9 expression through a related signaling pathway, which subsequently ensures the integrity of BBB function and reduces vascular permeability (28). So, it also indicates that non-M-PSCAs may develop into hemorrhagic MMD in the future. We may also conclude that higher expression of MMP-9 in vessel specimens have lower risk in postoperative CHP in MMD patients. However, some previous studies have shown that increased expression of MMP-9 in serum specimens have higher risk in postoperative CHP in MMD patients (29–31). It seems to be contradictory. However, previous studies were all check the MMP-9 level in serum rather than in vascular wall. The relationship between the expression of MMP-9 in intracranial vascular wall and postoperative CHP in MMD needs more studies to explore. As we mentioned, Suzuki stage 3 is the independent risk for M-PSCAs MMD, which means M-PSCAs MMDs are in a rapidly progressive stage and its collateral vessels will increase. Since MMD is a progressive disease, the level of MMP-9 is dynamic rather than stable during the disease progress. The relatively lower expression of MMP-9 in vascular specimens in M-PSCAs MMD, may leads to relatively low vascular permeability and a relatively complete functioning BBB. In order to pass through BBB, more MMP-9 in peripheral blood needs to be produced to promote angiogenesis in M-PSCAs MMD. Therefore, it may lead to this phenomena that M-PSCAs MMD has higher expression of MMP-9 in serum but lower in PSCAs vessel specimens. This needs to be further confirmed. On the other hand, the expression of MMP-9 in intracerebral arterial serum and peripheral blood serum in the M-PSCAs MMD may be lower than non-M-PSCAs MMD patients in this study, but M-PSCAs MMD patients still have higher risk postoperative CHP, which revealed that the hemodynamic sources from PSCAs is more important for postoperative CHP compared to vascular permeability caused by MMP-9. In future studies, we will further confirm the relationship between MMP-9 and CHP, hoping to provide reliable evidence for the treatment of MMD as well as preventing CHP.

All in all, this study still has certain limitations. First, our study focused on the tissue of the recipient PSCAs (distal MCA vessels) in adult MMD, and we have made some new research progress on adult MMD. However, for the treatment of pediatric MMD, we usually perform indirect bypass surgery, and it is challenging to obtain vascular specimens. Therefore, for the time being, this study is still not progressing on pediatric MMD. Then, the expression of MMP-9 and HIF-1*α* in vessel specimens were compared, but not in the serum. Therefore, we are unsure if there is a difference between in vessel specimens and serum. If there is, it may provide new findings. It is also worth exploring whether its expression in serum is more stable and accurate because of the tedious vascular immunohistochemical process. Lastly, the mechanism of postoperative CHP syndrome and PSCA thickness in patients with MMD needs to be further studied.

## Conclusions

Our results indicate that M-PSCAs MMD patients had thinner intima than non-MCAs MMD patients in the PSCAs. More importantly, HIF-1*α* and MMP-9 were overexpressed in PSCAs specimens in non-MCAs MMD.

## Data Availability

The original contributions presented in the study are included in the article/Supplementary Material, further inquiries can be directed to the corresponding author/s.
